# 1,3-Dimethyl-1*H*-indazol-6-amine

**DOI:** 10.1107/S1600536812013694

**Published:** 2012-04-04

**Authors:** Xiao-Kai Zhang, Bing-Ni Liu, Mo Liu, Deng-Ke Liu, Ping-Bao Wang

**Affiliations:** a Pharmaceutical College of Henan University, Henan Kaifeng 475000, People’s Republic of China; b Tianjin Institute of Pharmaceutical Research, Tianjin 300193, People’s Republic of China; c Tianjin Tnstitute of Pharmaceutical Rearch, Tianjin 300193, People’s Republic of China

## Abstract

The mol­ecular skeleton of the title compound, C_9_H_11_N_3_, is almost planar, with a maximum deviation of 0.0325 (19) Å for the amino N atom. In the crystal, N—H⋯N hydrogen bonds establish the packing.

## Related literature

For the synthesis of the title compound, see: Sorbera *et al.* (2006 ▶); Zhao *et al.* (2011 ▶). For related structures, see: Qi *et al.*(2010 ▶); Long *et al.* (2011 ▶). For the application of indazole derivatives in the synthesis of drugs, see: Collot *et al.* (1999 ▶).
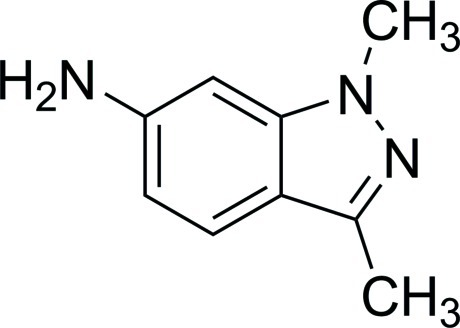


## Experimental

### 

#### Crystal data

C_9_H_11_N_3_
*M* *_r_* = 161.21
Orthorhombic, 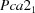

*a* = 18.3004 (10) Å
*b* = 8.3399 (7) Å
*c* = 5.6563 (1) Å
*V* = 863.28 (9) Å^3^
*Z* = 4
Mo *K*α radiation
μ = 0.08 mm^−1^
*T* = 293 K
0.22 × 0.18 × 0.12 mm

#### Data collection

Rigaku Saturn diffractometer
Absorption correction: multi-scan (*CrystalClear*; Rigaku/MSC, 2005 ▶) *T*_min_ = 0.983, *T*_max_ = 0.991
7967 measured reflections
2002 independent reflections
1588 reflections with *I* > 2σ(*I*)
*R*_int_ = 0.045

#### Refinement

*R*[*F* ^2^ > 2σ(*F* ^2^)] = 0.055
*wR*(*F* ^2^) = 0.149
*S* = 1.02
2002 reflections
118 parameters
4 restraints
H atoms treated by a mixture of independent and constrained refinement
Δρ_max_ = 0.16 e Å^−3^
Δρ_min_ = −0.14 e Å^−3^

### 

Data collection: *CrystalClear* (Rigaku/MSC, 2005 ▶); cell refinement: *CrystalClear*; data reduction: *CrystalClear*; program(s) used to solve structure: *SHELXS97* (Sheldrick, 2008 ▶); program(s) used to refine structure: *SHELXL97* (Sheldrick, 2008 ▶); molecular graphics: *SHELXTL* (Sheldrick, 2008 ▶); software used to prepare material for publication: *SHELXTL*.

## Supplementary Material

Crystal structure: contains datablock(s) global, I. DOI: 10.1107/S1600536812013694/kp2401sup1.cif

Structure factors: contains datablock(s) I. DOI: 10.1107/S1600536812013694/kp2401Isup2.hkl

Supplementary material file. DOI: 10.1107/S1600536812013694/kp2401Isup3.cdx

Supplementary material file. DOI: 10.1107/S1600536812013694/kp2401Isup4.cml

Additional supplementary materials:  crystallographic information; 3D view; checkCIF report

## Figures and Tables

**Table 1:** Hydrogen-bond geometry (Å, °)

*D*—H⋯*A*	*D*—H	H⋯*A*	*D*⋯*A*	*D*—H⋯*A*
N3—H3*A*⋯N1^i^	0.89 (1)	2.32 (1)	3.203 (2)	169 (2)
N3—H3*B*⋯N3^ii^	0.91 (1)	2.48 (1)	3.384 (2)	175 (2)

Symmetry codes: (i) 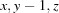; (ii) 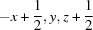.

**Fig. 1.**
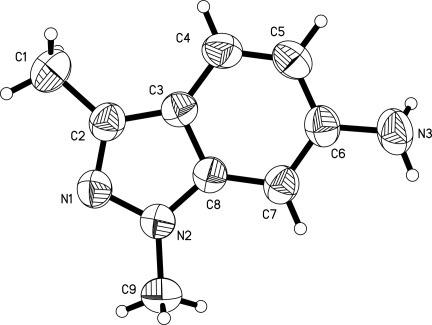
The molecular structure of (I), with the atom-numbering scheme and 50% probability displacement ellipsoids.

**Fig. 2.**
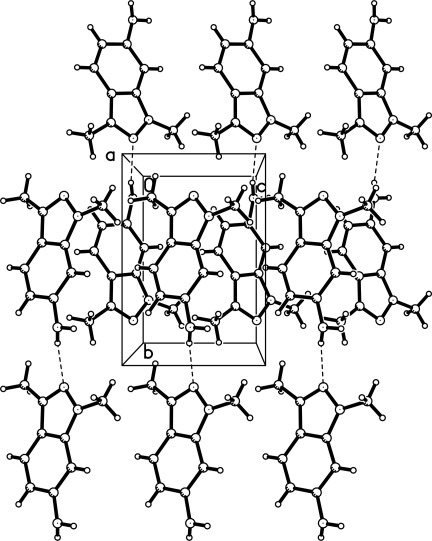
Packing diagram for (I) with hydrogen bonds (dashed lines).

## supplementary crystallographic information

### Comment

Some derivatives of indazole are important intermediates in the synthesis of drugs (Collot *et al.*1999). Here we report the crystal structure of the title compound(I).

In (I) the bond lengths and angles are normal and comparable with those reported for related compounds (Long *et al.*,2011; Qi *et al.*, 2010). The rings C3/C4/C5/C6/C7/C8 and C3/C2/N1/N2/C8 are almost coplaner forming a dihedral angle 0.82 (14)° (Fig. 1). The indazole ring system is almost planar with the maximal deviation of 0.0325 (19) Å for the atom N3. In the crystal structure intermolecular N–H···N hydrogen bonds (Fig. 2, Table 1) establish the packing.

### Experimental

Step 1: Dimethyl carbonate(7.5 g, 3 eq) was added to a solution of 3-methyl-6-nitro-1*H*-indazole(5 g, 1eq) and triethylene diamine(3.1 g, 1eq) in 15 mL DMF. After stirring of 10 h at 353 K, the mixture was poured into 150 mL cold water, after filtering and drying a mixture of 1,3-dimethyl-6-nitro-1*H*-indazole and 2,3-dimethyl -6-nitro-2*H*-indazole were obtained.1,3-Dimethyl-6-nitro-1*H*- indazole (2 g) was obtained by silicagel column chromatography.

Step 2: Pd/C(0.2 g) was added to a solution of 1,3-dimethyl-6-nitro-1*H*-indazole (2 g) in 10 mL ethanol. After the reaction system was kept in vacuum, the mixture was treated with continuous hydrogen stream. After stirring of 8 h, the reaction system was filtered to get yellow solution. The solution was left at room temperature, and colourless crystals were grown slowly.

### Refinement

C-bound H atoms were geometrically positioned (C—H 0.93–0.96 Å),and refined as riding with *U*_iso_=1.2–1.5*U*_eq_(C).

### Crystal data

**Table:**

C_9_H_11_N_3_	*F*(000) = 344
*M**_r_* = 161.21	*D*_x_ = 1.240 Mg m^−^^3^
Orthorhombic, *P**c**a*2_1_	Mo *K*α radiation, λ = 0.71073 Å
Hall symbol: P 2c -2ac	Cell parameters from 2241 reflections
*a* = 18.3004 (10) Å	θ = 3.3–27.9°
*b* = 8.3399 (7) Å	µ = 0.08 mm^−^^1^
*c* = 5.6563 (1) Å	*T* = 293 K
*V* = 863.28 (9) Å^3^	Prism, yellow
*Z* = 4	0.22 × 0.18 × 0.12 mm

### Data collection

**Table:**

Rigaku Saturn diffractometer	2002 independent reflections
Radiation source: rotating anode	1588 reflections with *I* > 2σ(*I*)
Confocal monochromator	*R*_int_ = 0.045
ω scans	θ_max_ = 27.8°, θ_min_ = 2.7°
Absorption correction: multi-scan (*CrystalClear*; Rigaku/MSC, 2005)	*h* = −23→24
*T*_min_ = 0.983, *T*_max_ = 0.991	*k* = −9→10
7967 measured reflections	*l* = −7→7

### Refinement

**Table:**

Refinement on *F*^2^	Secondary atom site location: difference Fourier map
Least-squares matrix: full	Hydrogen site location: inferred from neighbouring sites
*R*[*F*^2^ > 2σ(*F*^2^)] = 0.055	H atoms treated by a mixture of independent and constrained refinement
*wR*(*F*^2^) = 0.149	*w* = 1/[σ^2^(*F*_o_^2^) + (0.0907*P*)^2^] where *P* = (*F*_o_^2^ + 2*F*_c_^2^)/3
*S* = 1.02	(Δ/σ)_max_ = 0.002
2002 reflections	Δρ_max_ = 0.16 e Å^−^^3^
118 parameters	Δρ_min_ = −0.14 e Å^−^^3^
4 restraints	Extinction correction: *SHELXL97* (Sheldrick, 2008), Fc^*^=kFc[1+0.001xFc^2^λ^3^/sin(2θ)]^-1/4^
Primary atom site location: structure-invariant direct methods	Extinction coefficient: 0.12 (2)

### Special details

**Table:**

Geometry. All e.s.d.'s (except the e.s.d. in the dihedral angle between two l.s. planes) are estimated using the full covariance matrix. The cell e.s.d.'s are taken into account individually in the estimation of e.s.d.'s in distances, angles and torsion angles; correlations between e.s.d.'s in cell parameters are only used when they are defined by crystal symmetry. An approximate (isotropic) treatment of cell e.s.d.'s is used for estimating e.s.d.'s involving l.s. planes.
Refinement. Refinement of *F*^2^ against ALL reflections. The weighted *R*-factor *wR* and goodness of fit *S* are based on *F*^2^, conventional *R*-factors *R* are based on *F*, with *F* set to zero for negative *F*^2^. The threshold expression of *F*^2^ > σ(*F*^2^) is used only for calculating *R*-factors(gt) *etc*. and is not relevant to the choice of reflections for refinement. *R*-factors based on *F*^2^ are statistically about twice as large as those based on *F*, and *R*- factors based on ALL data will be even larger.

### Fractional atomic coordinates and isotropic or equivalent isotropic displacement parameters (Å^2^)

**Table:**

	*x*	*y*	*z*	*U*_iso_*/*U*_eq_	
N1	0.10351 (9)	0.83358 (18)	0.4784 (4)	0.0568 (5)	
N2	0.14711 (8)	0.72497 (19)	0.5968 (4)	0.0538 (5)	
N3	0.19922 (10)	0.1548 (2)	0.4581 (5)	0.0699 (6)	
H3A	0.1689 (11)	0.071 (2)	0.449 (5)	0.084*	
H3B	0.2287 (12)	0.150 (3)	0.587 (3)	0.084*	
C1	0.01866 (14)	0.8321 (3)	0.1420 (6)	0.0785 (8)	
H1A	−0.0293	0.7890	0.1680	0.118*	
H1B	0.0330	0.8131	−0.0188	0.118*	
H1C	0.0182	0.9454	0.1721	0.118*	
C2	0.07147 (10)	0.7530 (3)	0.3043 (5)	0.0549 (5)	
C3	0.09469 (10)	0.5900 (2)	0.3044 (4)	0.0493 (5)	
C4	0.08096 (11)	0.4531 (3)	0.1658 (5)	0.0596 (6)	
H4	0.0489	0.4579	0.0384	0.072*	
C5	0.11558 (12)	0.3130 (3)	0.2223 (5)	0.0615 (6)	
H5	0.1072	0.2228	0.1295	0.074*	
C6	0.16353 (10)	0.3008 (2)	0.4163 (5)	0.0559 (6)	
C7	0.17824 (11)	0.4336 (2)	0.5559 (4)	0.0529 (5)	
H7	0.2102	0.4277	0.6836	0.063*	
C8	0.14298 (9)	0.5768 (2)	0.4964 (4)	0.0472 (5)	
C9	0.19061 (12)	0.7763 (3)	0.7940 (5)	0.0624 (6)	
H9A	0.1936	0.6913	0.9081	0.094*	
H9B	0.1685	0.8688	0.8655	0.094*	
H9C	0.2388	0.8032	0.7402	0.094*	

### Atomic displacement parameters (Å^2^)

**Table:**

	*U*^11^	*U*^22^	*U*^33^	*U*^12^	*U*^13^	*U*^23^
N1	0.0555 (9)	0.0501 (9)	0.0648 (12)	0.0046 (6)	−0.0046 (8)	−0.0011 (9)
N2	0.0544 (8)	0.0497 (9)	0.0573 (10)	0.0024 (7)	−0.0077 (8)	−0.0037 (8)
N3	0.0716 (13)	0.0426 (9)	0.0956 (17)	−0.0012 (7)	−0.0004 (12)	0.0028 (11)
C1	0.0711 (14)	0.0782 (15)	0.0862 (19)	0.0149 (11)	−0.0214 (14)	0.0054 (14)
C2	0.0474 (9)	0.0563 (11)	0.0610 (12)	0.0026 (8)	−0.0019 (9)	0.0031 (10)
C3	0.0462 (9)	0.0521 (11)	0.0496 (11)	−0.0036 (7)	−0.0001 (8)	0.0017 (9)
C4	0.0581 (11)	0.0599 (12)	0.0608 (13)	−0.0082 (9)	−0.0086 (10)	−0.0051 (11)
C5	0.0655 (12)	0.0509 (11)	0.0682 (15)	−0.0097 (9)	−0.0008 (11)	−0.0082 (10)
C6	0.0517 (10)	0.0463 (10)	0.0696 (15)	−0.0031 (8)	0.0064 (10)	0.0027 (10)
C7	0.0509 (9)	0.0495 (11)	0.0581 (12)	−0.0016 (7)	0.0008 (9)	0.0074 (9)
C8	0.0429 (8)	0.0461 (10)	0.0526 (11)	−0.0025 (6)	0.0018 (8)	0.0037 (9)
C9	0.0663 (13)	0.0651 (13)	0.0556 (13)	−0.0020 (10)	−0.0097 (10)	−0.0054 (11)

### Geometric parameters (Å, º)

**Table:**

N1—C2	1.329 (3)	C3—C8	1.404 (3)
N1—N2	1.380 (2)	C3—C4	1.407 (3)
N2—C8	1.362 (2)	C4—C5	1.367 (3)
N2—C9	1.436 (3)	C4—H4	0.9300
N3—C6	1.401 (3)	C5—C6	1.409 (3)
N3—H3A	0.893 (9)	C5—H5	0.9300
N3—H3B	0.909 (10)	C6—C7	1.387 (3)
C1—C2	1.487 (3)	C7—C8	1.399 (3)
C1—H1A	0.9600	C7—H7	0.9300
C1—H1B	0.9600	C9—H9A	0.9600
C1—H1C	0.9600	C9—H9B	0.9600
C2—C3	1.424 (3)	C9—H9C	0.9600
			
C2—N1—N2	106.43 (17)	C5—C4—H4	120.6
C8—N2—N1	111.14 (17)	C3—C4—H4	120.6
C8—N2—C9	128.72 (17)	C4—C5—C6	122.2 (2)
N1—N2—C9	120.11 (17)	C4—C5—H5	118.9
C6—N3—H3A	112.3 (15)	C6—C5—H5	118.9
C6—N3—H3B	116.7 (15)	C7—C6—N3	120.5 (2)
H3A—N3—H3B	112.6 (15)	C7—C6—C5	120.41 (19)
C2—C1—H1A	109.5	N3—C6—C5	119.0 (2)
C2—C1—H1B	109.5	C6—C7—C8	117.1 (2)
H1A—C1—H1B	109.5	C6—C7—H7	121.4
C2—C1—H1C	109.5	C8—C7—H7	121.4
H1A—C1—H1C	109.5	N2—C8—C7	130.43 (19)
H1B—C1—H1C	109.5	N2—C8—C3	106.65 (16)
N1—C2—C3	110.53 (19)	C7—C8—C3	122.91 (18)
N1—C2—C1	121.3 (2)	N2—C9—H9A	109.5
C3—C2—C1	128.1 (2)	N2—C9—H9B	109.5
C8—C3—C4	118.65 (18)	H9A—C9—H9B	109.5
C8—C3—C2	105.24 (18)	N2—C9—H9C	109.5
C4—C3—C2	136.1 (2)	H9A—C9—H9C	109.5
C5—C4—C3	118.7 (2)	H9B—C9—H9C	109.5
			
C2—N1—N2—C8	0.5 (2)	N3—C6—C7—C8	177.1 (2)
C2—N1—N2—C9	178.8 (2)	C5—C6—C7—C8	0.7 (3)
N2—N1—C2—C3	−0.9 (2)	N1—N2—C8—C7	179.1 (2)
N2—N1—C2—C1	178.5 (2)	C9—N2—C8—C7	1.0 (3)
N1—C2—C3—C8	0.9 (2)	N1—N2—C8—C3	0.1 (2)
C1—C2—C3—C8	−178.4 (2)	C9—N2—C8—C3	−178.0 (2)
N1—C2—C3—C4	−178.9 (2)	C6—C7—C8—N2	−179.1 (2)
C1—C2—C3—C4	1.8 (4)	C6—C7—C8—C3	−0.2 (3)
C8—C3—C4—C5	−0.5 (3)	C4—C3—C8—N2	179.25 (18)
C2—C3—C4—C5	179.2 (2)	C2—C3—C8—N2	−0.6 (2)
C3—C4—C5—C6	1.0 (4)	C4—C3—C8—C7	0.2 (3)
C4—C5—C6—C7	−1.1 (3)	C2—C3—C8—C7	−179.67 (19)
C4—C5—C6—N3	−177.6 (2)		

### Hydrogen-bond geometry (Å, º)

**Table:**

*D*—H···*A*	*D*—H	H···*A*	*D*···*A*	*D*—H···*A*
N3—H3*A*···N1^i^	0.89 (1)	2.32 (1)	3.203 (2)	169 (2)
N3—H3*B*···N3^ii^	0.91 (1)	2.48 (1)	3.384 (2)	175 (2)

Symmetry codes: (i) *x*, *y*−1, *z*; (ii) −*x*+1/2, *y*, *z*+1/2.
